# Non-linear significant relationship between use of glycopeptides and isolation of vancomycin-resistant *Enterococcus species* in a university hospital setting

**DOI:** 10.1186/s13756-015-0064-5

**Published:** 2015-06-15

**Authors:** Christina Forstner, Magda Diab-Elschahawi, Danijel Kivaranovic, Wolfgang Graninger, Dieter Mitteregger, Maria Macher, Thomas Wrba, Elisabeth Presterl

**Affiliations:** Department of Medicine I, Division of Infectious Diseases and Tropical Medicine, Medical University of Vienna, Währinger Gürtel 18–20, 1090 Vienna Austria; Center for Infectious Diseases and Infection Control, Jena University Hospital, Jena, Germany; Department of Hospital Epidemiology and Infection Control, Medical University of Vienna, Währinger Gürtel 18–20, 1090 Vienna Austria; Section for Medical Statistics, Medical University of Vienna, Währinger Gürtel 18–20, 1090 Vienna Austria; Division of Clinical Microbiology, Department of Laboratory Medicine, Medical University of Vienna, Währinger Gürtel 18–20, 1090 Vienna Austria; Hospital Pharmacy, General Hospital of Vienna, Währinger Gürtel 18–20, 1090 Vienna Austria; Center of Medical Statistics, Informatics and Systems Intelligence, Medical University of Vienna, Währinger Gürtel 18–20, 1090 Vienna Austria

**Keywords:** Glycopeptides, Fluoroquinolones, Third generation cephalosporins, *Enterococcus faecium*, *Enterococcus faecalis*

## Abstract

**Background:**

Emergence of colonization and infection with vancomycin-resistant enterococci (VRE) has become a worldwide challenge. To investigate whether the increasing incidence of VRE isolation can be correlated with use of glycopeptides in the hospital setting, we conducted a hospital-wide two-year study in the university hospital of Vienna.

**Methods:**

Within the period from January 2011 through December 2012 all patients with isolation of invasive or non-invasive VRE were retrospectively included. Specialty-specific data concerning the consumption of vancomycin and teicoplanin, fluoroquinolones and third generation cephalosporins in defined daily doses (DDDs) from June 2010 through May 2012 were extracted from the hospital pharmacy computer system. To assess the relationship between the usage of those antibiotics and the incidence of VRE (VRE-rate per 10 000 patients) a Poisson regression was performed.

**Findings:**

In the study period 266 patients were colonized or infected with VRE. Specialty-specific VRE isolation was as follows: general surgical units (44 patients), bone marrow transplant unit (35 patients), general medical units (33 patients), cardiothoracic surgery (27 patients), nephrology (26 patients), haematooncology (22 patients), gastroenterology (17 patients), urology (17 patients), and the infectious diseases unit (11 patients). Hospital-wide consumption of glycopeptides was higher for teicoplanin than for vancomycin (26 242 versus 8677 DDDs). Specialty-specific VRE incidence significantly increased with the use of glycopeptides, fluoroquinolones or third generation cephalosporins (*p* < 0.001). The results of the Poisson regression for vancomycin (*p* = 0.0018) and teicoplanin (*p* < 0.0001) separately were both highly significant. Spearman’s correlation coefficient indicated a strong correlation between the two variables (rho = 0.8).

**Conclusion:**

Overall usage of glycopeptides, fluoroquinolones or third generation cephalosporins contributed to the emergence of VRE in the hospital setting.

## Introduction

Enterococci are part of the gastrointestinal bacterial flora and urogenital mucosa in healthy humans, but important nosocomial pathogens in a variety of invasive infections including endocarditis, bloodstream infections, wound infections, and meningitis [1, 2].

Colonization and infection with vancomycin-resistant enterococci (VRE), predominantly but not exclusively with *Enterococcus faecium*, are emerging worldwide [3–5]. The causes are multifactorial, but excessive usage of glycopeptides in animal husbandry and in human medicine may further enhance the incidence of VRE [6, 7]. According to the European Antimicrobial Resistance Surveillance Report (EARS), the incidence of invasive Austrian isolates of vancomycin-resistant *E. faecium* increased from 1.8 % in 2008 to 5.9 % in 2013 [5, 8]. Other neighbouring countries such as Germany reported an even more dramatic increase to 14.5 % of the proportion of invasive vancomycin-resistant *E. faecium* [8].

The aim of the present study was to investigate whether this increasing incidence of VRE isolation in our patients can be correlated with use of glycopeptides in the hospital setting.

## Methods

### Study location and study population

The General Hospital of Vienna, Austria, is a 2133–bed central hospital and the seat of the clinics of the Medical University of Vienna. Each year more than 100 000 patients receive inpatient treatment and a total of 1.25 million patients attend the outpatient clinics [9]. The university hospital is a renowned centre for solid organ transplantation - Europe-wide leading in lung transplantation-and haematooncology. After the study was approved by the local ethics committee of the Medical University of Vienna (EC No. 2004/2013), we retrospectively included all patients in the hospital with any isolation of VRE during the period of January 1, 2011 through December 31, 2012. The study was retrospective and observational with no interventions, therefore the need for informed consent was waived. The numbers of patients colonized or infected with VRE isolated from surveillance cultures and invasive cultures (from blood or other normally sterile body sites) were obtained from the hospital’s database (RDA/Archimed ALERT, Department of Infection Control and Hospital Epidemiology) for patients with multi-drug resistant pathogens.

In case of VRE screening selective enrichment media (brain-heart infusion containing vancomycin and ceftazidime as well as chromID VRE, bioMérieux, Marcy l’Etoile, France) were inoculated, while clinical samples for non-targeted culture were processed following standard procedures dependent on the type of specimen, i.e. non-selective enrichment broth (brain-heart infusion) was included only in case of material from primary sterile body sites. *Enterococcus faecalis* was identified by the formation of black colonies on tellurit agar plates, while other *Enterococcus* species were identified by VITEK 2 GP (bioMérieux) or MALDI-TOF mass spectrometry (Bruker Daltonics GmbH, Bremen, Germany). If the vancomycin and teicoplanin disc diffusion test according to the current version of the EUCAST methodology [10] displayed glycopeptide resistance, E tests for both substances following the manufacturer’s instructions (bioMérieux) were performed. Presence of the VanA or VanB phenotype was deduced from the observed minimal inhibitory concentrations of vancomycin and teicoplanin [11].

For avoidance of nosocomial transmissions, a targeted risk adjusted screening policy with subsequent isolation (single room or, if not possible, strict contact isolation) of patients colonized or infected with VRE is implemented at our institution.

### Antibiotic usage

Glycopeptides, fluoroquinolones and third generation cephalosporins were classified according to the anatomical therapeutic chemical (ATC) system. In our institution the following agents were used: vancomycin, teicoplanin, ciprofloxacin, levofloxacin, moxifloxacin, ceftriaxone, cefotaxime, ceftazidime and cefixime. Hospital-wide and specialty-specific data on consumption of those antibiotics were derived from the hospital pharmacy for the period from June 1, 2010 through and including May 31, 2012. Antibiotic usage was measured in defined daily doses (DDDs). In accordance with the ATC classification/DDD index 2013, the glycopeptide DDDs used were vancomycin 2 g and teicoplanin 0.4 g, the fluoroquinolone DDDs used were levofloxacin 0.5 g, moxifloxacin 0.4 g and ciprofloxacin 1 g for oral administration or 0.5 g for parenteral administration, the third generation cephalosporin DDDs used were ceftriaxone 2 g, cefotaxime 4 g, ceftazidime 4 g and cefixime 0.4 g [12].

### Statistical analysis

In order to assess the relationship between the antibiotic usage and the incidence of VRE (VRE-rate per 10 000 patients), a Poisson regression model was fitted with the number of VRE-positive patients as the dependent variable, logarithm of the mean usage of glycopeptides, fluoroquinolones or third generation cephalosporins as independent variable and the total number of patients in each unit as an offset.

As in a previous surveillance study by Kritsotakis *et al.* a dynamic relationship between antimicrobial use of glycopeptides and an increase in the VRE incidence was identified with average delays between 2 and 6 months [13], we selected to compare time-delayed 2-year periods of 6 months between antibiotic consumption and VRE isolation.

Model diagnostics revealed that the non-transformed mean usage did not fit the data well due to a high leverage point (influential point). Further to control for possible violations of the distribution assumptions, White’s robust covariance estimator was calculated [14]. Z-tests were performed using the coefficients of the Poisson model and the robust standard errors. *P*-values of ≤0.05 were considered to be statistically significant. All calculations were performed using R 3.0.2.

## Results

In the pre-study period from 2004 through 2010, a continuous increase in the number of VRE-positive patients was detected in the hospital, from a single documented case of VRE in 2004 (corresponding to 0.001 patients per 10 000 admissions), 67 cases of VRE in 2007 (corresponding to 0.066 patients per 10 000 admissions) to a peak of 129 VRE-positive patients in 2010 (corresponding to 0.125 patients per 10 000 admissions).

During the subsequent two-year study period, a total of 99 635 patients were admitted to the hospital in 2011 and 102 083 in 2012. The overall VRE incidence was 0.131 per 10 000 admissions in the first year and 0.156 per 10 000 admissions in the second year. Among these patients, 261 patients were colonized or infected with a single VRE species (*E. faecium,* n = 258; *E. faecalis*, n = 3), and five patients were colonized with 2 VRE species (*E. faecium,* n = 5; *E. faecalis*, n = 4; *E. raffinosus*, n = 1). The VanA phenotype was detected in the VRE isolates of 234 (88 %) patients and VanB phenotype in 33 (12 %) patients.

As shown in the Table 1, most patients were only colonized by non-invasive VRE-isolates, which were isolated from screening cultures (faeces or rectal swab, followed by skin swab, oropharyngeal swab). Among 87 patients with proven VRE-infection, 18 patients developed VRE bacteraemia with *E. faecium*.

**Table 1:** Hospital-wide trends in colonizing and invasive vancomycin-resistant *Enterococcus* (VRE) species during the study period 2011 through 2012

Species	Annual total no. (%) patients with VRE		Annual no. (%) patients only colonized with VRE		Annual no. (%) patients infected with VRE	
	2011	2012	2011	2012	2011	2012
Any *Enterococcus* sp.	123 (100)	143 (100)	87 (71)	92 (64)	36 (29)	51 (36)
*E. faecium*	123 (100)	140 (98)	87 (71)	90 (63)	36 (29)	50 (35)
*E. faecalis*	3 (2.4)	4 (2.8)	3 (2.4)	2 (1.4)	0	2 (1.4)
*E. raffinosus*	1 (0.8)	0	1 (0.8)	0	0	0

In case of isolation of more than one *Enterococcus spp.* in one patient, the patient was only counted once for the category any *Enterococcus sp.*

**Fig. 1:**
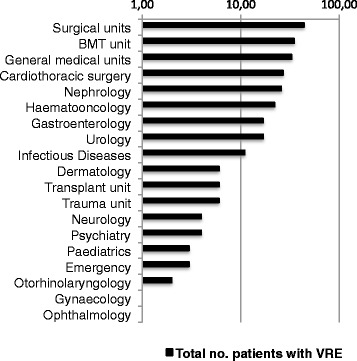
Specialty-specific isolation of VRE in the General Hospital of Vienna during the two-year study period

A total of 201 patients were admitted to inpatient wards, 52 patients to intensive care units and 13 were outpatients. The specialties most frequently involved were general surgical (44 patients), followed by the bone marrow transplantation (BMT) unit (35 patients), general medicine (33 patients), cardiothoracic surgery (27 patients), nephrology (26 patients), haematooncology (22 patients), gastroenterology (17 patients), urology (17 patients), and the infectious diseases unit (11 patients) (Fig. 1).

**Fig. 2:**
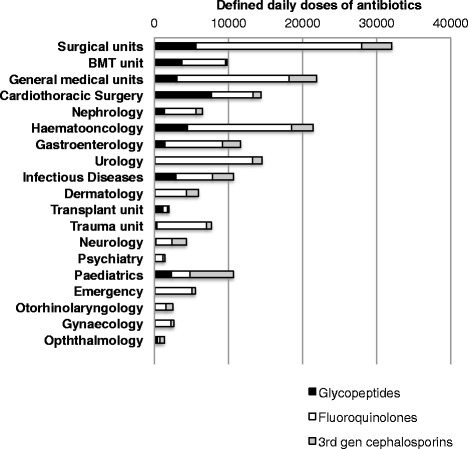
Specialty-specific usage of glycopeptides, fluoroquinolones and third generation cephalosporins in the General Hospital of Vienna during the period June 2010 to June 2012

The total hospital-wide numbers of antimicrobial DDDs were as follows:

8677 for vancomycin, 26 242 for teicoplanin, 64 788 for ciprofloxacin, 32 264 for levofloxacin, 22 155 for moxifloxacin, 13 080 for ceftriaxone, 10 910 for cefotaxime, 6958 for ceftazidime, and 1651 for cefixime. Total consumption of glycopeptides, fluoroquinolones and third generation cephalosporins per specialty is shown in Fig. 2.

**Fig. 3:**
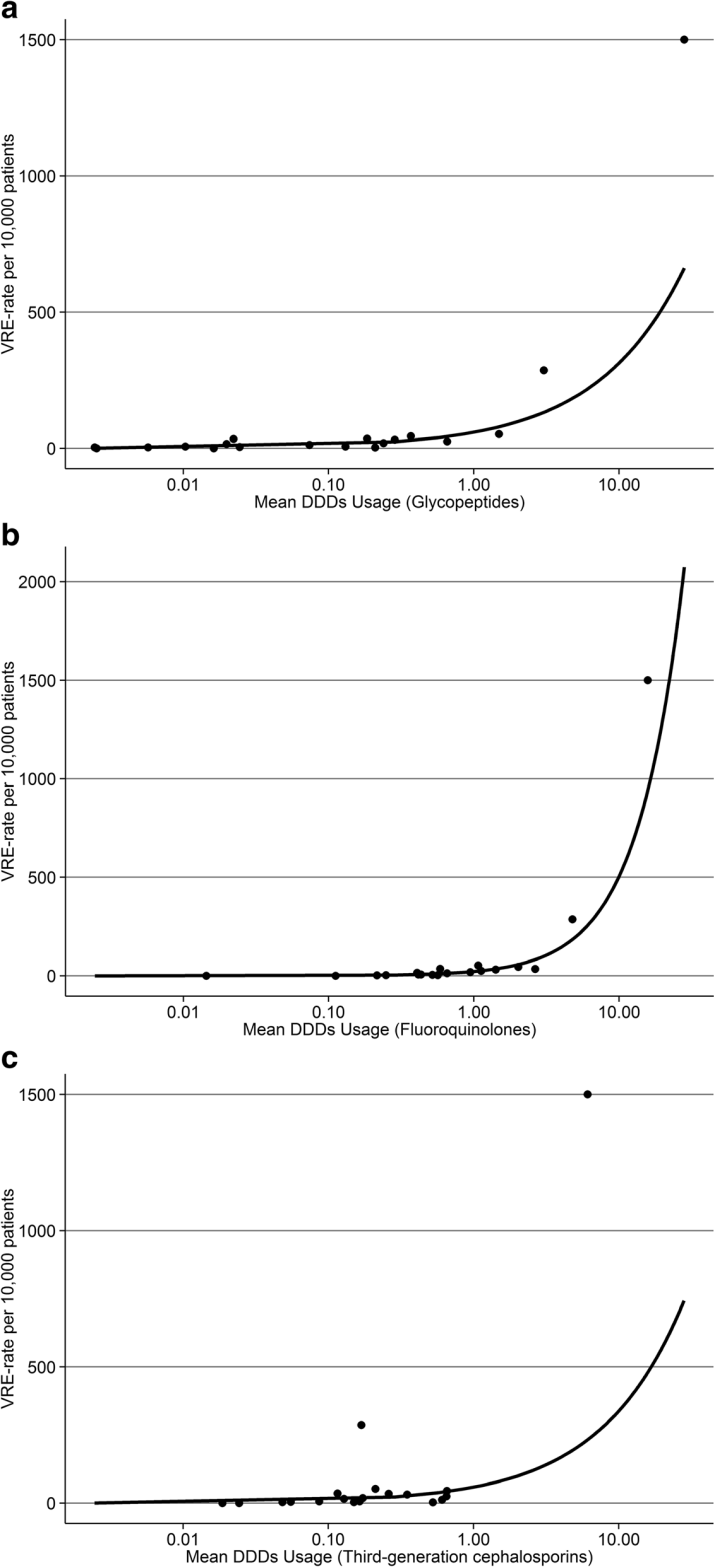
Non-linear relationship between the usage of glycopeptides (**a**), fluoroquinolones (**b**) or third generation cephalosporins (**c**) and VRE-rate per 10 000 patients. The points indicate observations and the solid line the fit of the Poisson regression model

Specialty-specific VRE incidence significantly increased with the use of glycopeptides, fluoroquinolones or third generation cephalosporins (*p* < 0.001). Figure 3 shows a non-linear relationship with a dramatic increase in the incidence of VRE with high glycopeptide, fluoroquinolone or third generation cephalosporin usage. The results of the Poisson regression for vancomycin (*p* = 0.0018) and teicoplanin separately (*p* < 0.0001) were both highly significant. Spearman’s correlation coefficient indicated a strong correlation between the two variables (rho = 0.8).

## Discussion

In the present study we identified a positive non-linear relationship between the specialty-specific isolation of VRE and total glycopeptide, fluoroquinolone or third generation cephalosporin consumption. Most patients with VRE-positive cultures were simply colonized, but 6.8 % experienced bloodstream infection caused by vancomycin-resistant *E. faecium*. Despite less clinical relevance of VRE compared to infections caused by methicillin-resistant *Staphylococcus aureus* (MRSA), management of invasive VRE infections is challenging because of limited treatment options. Furthermore, enterococcal bacteraemia was associated with increased risk of death in allogeneic haematopoietic stem cell transplant recipients [15]. During a 7-year surveillance study, a dynamic relationship was identified between antimicrobial use of glycopeptides, fluoroquinolones, and extended-spectrum cephalosporins and an increase in the incidence of VRE, with average delays between 2 and 6 months [13]. In contrast, other studies do not report such a direct relationship between glycopeptide use and VRE isolation [16] but have shown an association between a higher incidence of VRE-bloodstream infection and previous hospital use of other, non-glycopeptide, antimicrobials such as the third-generation cephalosporin ceftriaxone [17]. In a recent systematic review it was not possible to conclusively determine a potential role for reduction of vancomycin use in controlling VRE [18]. But, in a retrospective case–control study in a university hospital of Korea, vancomycin use significantly prolonged the duration of VRE carriage among intensive care patients already colonized with vancomycin-resistant *E. faecium* [19]. However, because of the high tenacity of VRE in the environment, measures other than reduction of glycopeptide use, such as isolation of patients and enforced environmental cleaning, are necessary to control its spread [20]. Patients who are at risk are consequently screened at admittance and isolated in single rooms. We also hypothesize that in our hospital the departments most frequently involved treat patients with chronic diseases for a long period of time, and VRE emerge due to glycopeptide use.

Vancomycin and teicoplanin are used therapeutically in our hospital to treat invasive infections with gram-positive multi-drug resistant microorganisms or as empirical therapy in severe sepsis or septic shock in intensive care and immunosuppressed patients. During the period 2000 through 2011, vancomycin was used most frequently in our institution as first-line treatment in patients with MRSA bacteraemia, followed by teicoplanin [21].

Furthermore, in patients with moderate to severe colitis caused by *Clostridium difficile,* oral glycopeptides can promote acquisition of gastrointestinal VRE [22], whereas the use of other oral antimicrobial agents against *C. difficile*, such as fidaxomicin, decrease the risk of VRE acquisition [23].

A limitation of our study was that we could not assess the impact of oral versus intravenous use of glycopeptides on the incidence of VRE separately. During the observation period, only the intravenous formulations of vancomycin and teicoplanin were provided for oral use, because no oral formulations were available.

## Conclusions

We demonstrated a positive non-linear relationship between the specialty-specific isolation of VRE and glycopeptide, fluoroquinolone or third generation cephalosporin usage in the University Hospital of Vienna. Although VRE incidence is increasing in our institution, the increase remains moderate, and is probably controlled by infection control measures. Nevertheless, to minimize the spread of glycopeptide-resistant *Enterococcus* species in the hospital setting, the implementation of a program for prudent use of glycopeptides, fluoroquinolones and third generation cephalosporins is warranted.
